# Difficulties in The Dietary Management of a Girl with Two Diseases Requiring a Special Diet

**DOI:** 10.34763/devperiodmed.20182203.225228

**Published:** 2018-10-04

**Authors:** Agnieszka Kowalik, Danuta Gajewska, Jolanta Sykut-Cegielska

**Affiliations:** 1 Department of Inborn Errors of Metabolism and Paediatrics, Institute of Mother and Child, Warsaw, Poland; 2 Department of Dietetics, University of Life Sciences, Warsaw, Poland

**Keywords:** 3 – methylcrotonylglycinuria, diabetes mellitus, dietary treatment, 3-metylokrotonyloglicynuria, cukrzyca, leczenie dietetyczne

## Abstract

3-Methylcrotonylglycinuria (3-MCG) is an autosomal recessive inborn error of leucine metabolism caused by the deficiency of 3-methylocrotonyl-CoA carboxylase (3-MCC deficiency). It is the most commonly detected organic aciduria in newborn screening conducted by tandem mass spectrometry (MS/MS) [1, 2]. The clinical phenotype is heterogeneous, ranging from asymptomatic to acute metabolic decompensations [3, 4]. Although at least in severe cases and in acute life threatening episodes limiting natural protein intake (particularly leucine) together with high caloric intake during catabolic periods is required, the need for specific dietary management often seems questionable [2]. In contrast with the 3-MCC deficiency, in diabetes mellitus type 1 (DM1) a diet based on carbohydrate and protein-fat exchangers is beyond dispute. However, as DM1 is quite a common disease, it may occur in a single patient with a rare disease, such as 3-MCC deficiency.

## Streszczenie

3-metylokrotonyloglicynuria (ang. 3-methylcrotonylglycinuria, 3-MCG) jest dziedziczoną autosomalnie recesywnie wrodzoną wadą metabolizmu (wwm) leucyny, spowodowaną deficytem karboksylazy 3-metylokrotonylo- CoA. Jest najczęściej wykrywaną acydurią organiczną w skriningu noworodkowym prowadzonym metodą tandemowej spektrometrii mas (MS/MS) [1, 2]. Kliniczny fenotyp jest bardzo zróżnicowany, od bezobjawowego po ostrą dekompensację metaboliczną [3, 4]. Chociaż przynajmniej w przypadkach o ciężkim przebiegu i w epizodach zagrażających życiu – jest wymagane ograniczenie białka naturalnego (zwłaszcza leucyny) razem z podażą wysokokaloryczną podczas stanów katabolizmu, potrzeba wprowadzenia specjalnego leczenia dietetycznego często wydaje się być wątpliwa [2]. W przeciwieństwie do 3-MCG stosowanie diety w cukrzycy typu 1 (ang. diabetes mellitus, DM1) opartej na wymiennikach węglowodanowych i białkowo-tłuszczowych nie podlega dyskusji. Ze względu na częstsze występowanie cukrzycy typu 1 może się zdarzyć, że dwie takie choroby jak deficyt 3-MCC i DM1 zostaną rozpoznane u jednego dziecka.

We report on an 8-year-old girl suffering from 3-MCC deficiency and DM1. Her birth weight was 3650 g and she was exclusively breastfed for 6 months. Up to 2 years of age she grew properly, without any alarming symptoms. At the age of 3 years and 4 months she was admitted to hospital due to hyperglycemia (500 mg/dL) and severe ketoacidosis. Over the last 9 months before admission to hospital the lack of weight gain, polyuria and polydipsia were observed. Due to such typical signs and symptoms she was diagnosed with DM1; her level of HbA1c reached 9.3%. Standard insulin therapy was initially started, in the form of a continuous intravenous infusion at a dose of 0.2-1.0 units/hour for 37 hours. After 8 hours of the infusion, oral nutrition was introduced, based on the carbohydrate exchange system. From the third day of hospitalization, subcutaneous injections of insulin (Humalog and Humulin N) were started. One day later, unexplained vomiting was observed. During the reinterview, the child’s mother mentioned the diagnosis of 3-MCC deficiency during newborn screening and shared the information which she received about this disease. As there were no symptoms of the disease, no dietary modifications had been recommended until that time. Therefore, during hospitalization, urine organic acid analysis, by means of the GC-MS method was ordered and revealed an excessive increase of 3-hydroxyisovaleric acid, 3-methylcrotonylglycine and severe ketonuria. This confirmed the diagnosis of the 3-MCC deficiency. Subsequent biochemical analysis showed a high level of plasma glutamine (951 μmol/L) and a decreased concentration of free and total carnitine (12 and 24 μmol/L, respectively). Consequently, in addition to the diet based on the carbohydrate exchange system, the medical nutrition therapy for 3-MCC deficiency was introduced [5]. The intake of natural protein, including leucine (up to 70-40 mg /kg bw), was reduced. The protein replacement leucine-free formula at a dose of 10 g/day was added to the diet. Oral supplementation with L-carnitine at a dose of 300 mg per day was introduced. After obtaining the normalisation of biochemical parameters, the patient was discharged home.

At the age of 4 years and 5 months the girl was admitted to hospital due to metabolic decompensation caused by a gastro-intestinal infection. Her treatment began with a continuous infusion of 10% glucose, initially at a rate of 80 ml/h and then 55 ml/h, so the total daily energy intake (from food and intravenous glucose) amounted to 1200-1300 kcal. After the disappearance of vomiting and the return to oral nutrition, recommendations prior to infection were applied again. Information about the current difficulties in the implementation of dietary recommendations, especially regarding the requirements for 3MCC deficiency (refusal of formula intake) were obtained from the interview with the child’s mother.

The parents were afraid of hyperglycemia, hence they did not follow the recommendations on the use of leucine-free formula in 2-3 doses dissolved in fruit juice. Also, the increase in the calorie intake during the infection, recommended due to 3MCC deficiency, was considered by the parents a risk of the exacerbation of diabetes. Re-training for the parents was conducted in order to explain the principles of the management of each of the two disorders. Table I shows the content of energy, natural protein, the equivalent of protein and leucine in the diet of the girl during the observation period.

**Table I:** Energy, protein and leucine intake in different periods of dietary management. Tabela I. Wartość energetyczna, spożycie białka i leucyny w różnych okresach leczenia dietetycznego.

Diet in different treatment periods *Dieta w różnym okresie leczenia*	Age [years] *Wiek [lata]*	Body weight [kg] *Masa ciała [kg]*	Energy [kcal/day] *Energia [kcal/dzień]*	Total protein [g/day] *Białko całkowite [g/dzień]*	Natural protein [g/kg bw] *Białko naturalne [g/kg m.c]*	Protein equivalent [g/kg bw] *Białko ekwiwalent [g/kg m.c]*	Leucine [mg/kg bw] *Leucyna [mg/kg m.c]*
before leucine restriction *przed ograniczeniem leucyny*	34/12	17.6	1250	30	1.7	0	85
with decreased amount of natural *protein z obniżoną ilością białka naturalnego*	35/12	17.9	1300	23	1.3	0	65
with leucine-free formula z preparatem bez *leucyny*	3 7/12	19.1	1300	30	1.17	0.4	60
during metabolic decompensation *podczas dekompensacji metabolicznej*	45/12	22	520 kcal (oral) + 768 kcal from infusion of 10% glucose. then 1000 kcal (oral) 520 *kcal (doustnie) + 768 kcal z wlewu 10% glukozy. następnie 1000 kcal (doustnie)*	22	0.8	0.2	40
in metabolic balance *w równowadze metabolicznej*	55/12	23	1300	33	1.0	0.43	50

## Discussion

Early detection of inborn errors of metabolism (IEM) by newborn screening ensures that appropriate treatment is conducted early enough to prevent serious complications. It happens that the parents of children with 3-MCC deficiency, detected in NBS, dispute the validity of the diagnosis and the treatment when observing the asymptomatic disease and normal development of the baby. The importance and the consequences of parental noncompliance with the recommended treatment for a 19-month old girl with 3-MCC deficiency were described by Ficicioglu and Payan [6]. In the case described above, the parents’ lack of understanding of the risks brought on by the disease and the differences in dealing with situations that are often initially asymptomatic was the cause of acute metabolic decompensation. The coexistence of two genetically determined diseases in a single patient is rarely recognised. Burlina et al. [7] reported a case of a boy diagnosed with DM1 at the age of 26 months, who presented with impaired walking, dysphagia and axial hypotonia identified 6 months later during an acute infection caused by glutaric aciduria type 1.

The dietary treatment of inborn errors of metabolism, including 3-MCC deficiency, requires limiting the consumption of many foods, including high-protein food. For children aged 4-7, a safe intake of leucine ranges from 35 to 65 mg/kg bw. A supplementation with a mixture of essential (excluding leucine), and nonessential amino acids to maintain a positive balance of protein and to provide optimal growth and development is necessary [5]. The dietary imbalance described in the case reported could lead to the lack of metabolic control, and in the long-term could negatively affect the patient’s development. In order to reach the adequate amount of leucine in everyday diet, knowledge of the food composition and the calculation of the nutritional value of the total diet is necessary. The recommendations in 3-MMC deficiency and DM1 are quite opposite, at least in catabolic conditions. In 3-MCC deficiency, there is a need for high caloric intake, so intravenous and oral glucose is necessary, but in the DM1 – such management should be avoided. The course of the illness and the prognosis for the future depend on the good cooperation between the parents of the child with IEM and the medical team, or also the dietitian, in case a specific diet is required.

## Conclusion

This study demonstrates that two diseases requiring a specific dietary management diagnosed in one child may be a huge challenge for both the medical team, as well as the child’s parents. The authors show a case report of a patient with two coexisting diseases – 3-MCC deficiency and DM1. Difficulties in the dietary management of such a patient require regular, long-term monitoring with close cooperation between the patient’s parents and a dietitian.
